# Alcohol outlets and clusters of violence

**DOI:** 10.1186/1476-072X-10-30

**Published:** 2011-05-04

**Authors:** Tony H Grubesic, William Alex Pridemore

**Affiliations:** 1 Geographic Information Systems and Spatial Analysis Laboratory, College of Information Science and Technology, Drexel University, PA 19104, Philadelphia; 2 Department of Criminal Justice, Indiana University, IN 47405, Bloomington

## Abstract

**Background:**

Alcohol related violence continues to be a major public health problem in the United States. In particular, there is substantial evidence of an association between alcohol outlets and assault. However, because the specific geographic relationships between alcohol outlets and the *distribution *of violence remains obscured, it is important to identify the spatial linkages that may exist, enhancing public health efforts to curb both violence and morbidity.

**Methods:**

The present study utilizes police-recorded data on simple and aggravated assaults in Cincinnati, Ohio. Addresses of alcohol outlets for Cincinnati, including all bars, alcohol-serving restaurants, and off-premise liquor and convenience stores were obtained from the Ohio Division of Liquor Control and geocoded for analysis. A combination of proximity analysis, spatial cluster detection approaches and a geographic information system were used to identify clusters of alcohol outlets and the distribution of violence around them.

**Results:**

A brief review of the empirical work relating to alcohol outlet density and violence is provided, noting that the majority of this literature is cross-sectional and ecological in nature, yielding a somewhat haphazard and aggregate view of how outlet type(s) and neighborhood characteristics like social organization and land use are related to assaultive violence. The results of the statistical analysis for Cincinnati suggest that while alcohol outlets are not problematic per se, assaultive violence has a propensity to cluster around agglomerations of alcohol outlets. This spatial relationship varies by distance and is also related to the characteristics of the alcohol outlet agglomeration. Specifically, spatially dense distributions of outlets appear to be more prone to clusters of assaultive violence when compared to agglomerations with a lower density of outlets.

**Conclusion:**

With a more thorough understanding of the spatial relationships between alcohol outlets and the distribution of assaults, policymakers in urban areas can make more informed regulatory decisions regarding alcohol licenses. Further, this research suggests that public health officials and epidemiologists need to develop a better understanding of what actually occurs in and around alcohol outlets, determining what factors (whether outlet, neighborhood, or spatially related) help fuel their relationship with violence and other alcohol-related harm.

## Background

This study took advantage of proximity analysis and spatial cluster detection to understand better the spatial relationship between agglomerations of alcohol outlets and levels of assault in urban areas. Several studies from the disciplines of criminology, epidemiology, sociology, public health, and geography have found an association between alcohol outlet density and violence rates [1-7]. Some studies in this genre examine the *characteristics of bars *that might put them at higher risk for hosting violence - for example, those that promote irresponsible serving practices and binge drinking [8], that are poorly designed internally and thus create crowded spaces [9], and that possess other problematic characteristics [10-12]. Other studies take a different approach, however, focusing on the strength of the ecological association between outlet density and assault and how it may be moderated by *neighborhood characteristics *like socioeconomic disadvantage [2,13,14], social organization [15], and land use [16].

The latter studies are of interest to us, as they examine the characteristics of spaces (e.g., neighborhoods, census tracts, block groups) that are associated with higher rates of crime and violence in those spaces. However, while these analyses consistently find a positive ecological association between alcohol outlet density and assault rate, they do not provide an understanding of how violence is geographically distributed around outlets and clusters of outlets. At most these studies usually view spatial relationships like autocorrelation as a nuisance and control for it in their models. This is understandable, because these prior studies addressed different theoretical questions than we address here. Yet if we wish to understand better the association between clusters of outlets and clusters of violence, we must now move beyond simply confirming the ecological association between the two and learn more about the spatial nature of that relationship, which is where geographic analytical techniques can be of tremendous benefit. One example of the type of work that does employ spatial analytical techniques to more closely examine the spatial relationship between outlets and assaults was Murray and Roncek [17], who revealed that the conclusions drawn about this association may differ depending upon whether analysts employ Euclidian radial buffers or adjacency techniques. Similarly, though not examining alcohol outlet density, spatial criminologists Andresen and Malleson [18] recently employed spatial point pattern tests to determine if spatial patterns found at higher units of analysis (e.g., census tracts) held at lower levels (e.g., dissemination areas and street segments). The authors found that while the general patterns held, there was substantial variation within the larger units.

Together with what we know from the geography literature about theory and method, the findings from Murray and Roncek [17], Andresen and Malleson [18], and others [19,20] reveal the importance of examining the *spatial nature of the relationship *between alcohol outlets and violence, not simply whether or not such an ecological association exists. Further, examining these relationships at lower levels of aggregation may not only reveal greater detail about the nature of the relationship, but provide practical information about how general alcohol policy or specific decisions about granting individual licenses should be made, especially when taking into account what we already know about how neighborhood characteristics moderate the association. Therefore, in the present study we apply specific spatial analytical techniques - proximity analysis and spatial cluster detection - to go beyond the simple outlet density-violence ecological association to search for agglomerations of alcohol outlets and then to determine not only if these agglomerations are sites of heightened risk of assaults but also the distance over which these agglomerations exert their influence.

## Methods

### Study Area and Data

Cincinnati, Ohio, had a population of about 334,000 residents and a violent crime rate of 1,079 per 100,000 residents in 2008. The latter is similar to several other large US cities. The unit of analysis for this paper was the Census block. It is important not to confuse blocks with block groups. Census blocks are the smallest units of geography that the Census Bureau makes demographic data publicly available, and spatial analysts of crime are increasingly recognizing the importance of examining small units of analysis [18,21]. On average, there are approximately 39 census blocks *in *a block group. For the city of Cincinnati, blocks average 0.02 square miles.

The Cincinnati Police Department provided data on all crimes reported to the police between January and June of 2008. Each record included the Uniform Crime Report (UCR) code, time and date of the offense, address, and description of the location (e.g., street, single family house, etc.). We selected simple assaults (*n *= 2,298) and aggravated assaults (*n *= 479) for further analysis. The UCR defines simple assaults as those that do not involve a firearm, knife, cutting instrument, or other dangerous weapon, and in which the victim did not sustain serious injuries. The UCR defines aggravated assault as an unlawful attack by one person upon another for the purpose of inflicting severe or aggravated bodily injury. These assaults are usually accompanied by the use of a weapon or other means likely to produce death or great bodily harm.

Assault data were geocoded using the Centrus geocoding engine [22]. Only events assigned a street-level match were utilized for analysis. In effect, these are "rooftop" hits, where the geocoded point is placed on the rooftop of the structure associated with the input address. This is the best match possible from a geocoding algorithm. Approximately 95% of the original assault data utilized for this analysis were successfully geocoded. Note that, as with all studies related to violence and alcohol outlet density, these are all assaults and not only assaults that are "alcohol-related," as police data on the latter are unreliable. Once all of the recorded assaults were assigned supplementary spatial information (i.e., geocoded), they were aggregated to blocks (*n *= 3,880). The distribution of simple assaults (*n *= 2,297) in the blocks ranged from 0 (*n *= 2,929) to 43 in a single block, with a mean of 1. The distribution of aggravated assault (*n *= 479) ranged from 0 to 12, with a mean of 0. Finally, the total population at risk was 334,264, the average simple assault frequency was 0.00687, and the average aggravated assault frequency was 0.00142. For more details on the frequency distributions and descriptive statistics regarding simple and aggravated assaults, see Tables 1 and 2.

**Table 1:** Simple Assault Distributions by Block and Associated Descriptive Statistics

Assault Count	Frequency by Block	Cumulative Percent
0	2929	75.5
1	474	87.7
2	222	93.4
3	98	96
4	59	97.5
5	30	98.2
6	18	98.7
7	14	99.1
8	10	99.3
9+	26	100

*N*	3880	
Mean	0.59	
Median	0	
Standard Deviation	1.805	
Variance	3.258	

**Table 2:** Aggravated Assault Distributions by Block and Associated Descriptive Statistics

Assault Count	Frequency by Block	Cumulative Percent
0	3523	90.8
1	282	98.1
2	50	99.4
3	20	99.9
4	1	99.9
5	1	99.9
6	1	99.9
8	1	100
12	1	100

*N*	3880	
Mean	0.12	
Median	0	
Standard Deviation	0.479	
Variance	0.229	

Alcohol outlet data were obtained from the Ohio Division of Liquor Control [23]. These data include permit designations that allowed us to disaggregate by outlet type, including off-premise outlets, restaurants and bars. For our purposes, off-premise outlets were those with C1, C2, and C2X licenses, restaurants were D1, D2, and D2X, and bars were D3, D5, and D6. In Ohio, liquor and grocery stores that sell spirits have no Number-Letter license, but instead are referred to as "liquor agencies" and go through a different application process. There are 14 of these in Cincinnati and we counted them as "off-premise." There were 683 unique outlets in Cincinnati during the summer of 2008, and it is common for a single outlet to operate under more than one permit (e.g., C1 and C2). Each outlet was geocoded using the same process described above and aggregated to blocks. Again, approximately 95% of the outlets were successfully geocoded using the Centrus engine. The remaining 5% of the outlets were manually assigned geographic coordinates using a cadastral (i.e., parcel) database from Hamilton County, Ohio. It is also important to note that the outlet premise addresses were utilized in the geocoding algorithm and the manual parcel matching process, rather than the addresses associated with the licensee.

### Cartographic Analysis

Basic cartographic analysis is conducted for exploring the spatial distribution of assaultive violence and alcohol outlets for Cincinnati. In addition to choropleth mapping, simple metrics associated with violence risk, by block, are calculated and visualized. One approach for evaluating the degree of risk associated with the distribution of assaultive violence is a proportional measure of total assaults (simple or aggravated) in a region, *A_i_*, and the total population within the region, *P_i_*. The raw rate can then be represented by a simple proportion, *r*_*i *_= *A*_*i*_/*P*_*i*_. Although raw rates and risk are not particularly informative, relative risks can also be captured by comparing the rate at each location to the overall mean, which is a ratio of all simple or aggravated assaults in Cincinnati over the total population:(1)

Where *n *is the number of enumeration districts (e.g., blocks) in the study region. Finally, based on this measure of average risk, an expected distribution of assaults can also be derived from the underlying population, . Using this expected distribution, if the number of assaults either exceeds or falls short of the expected number for a location, a measure of excess risk is captured.

### Modeling

In an effort to better define agglomerations of alcohol outlets and their associated locations, the local Moran's *I *statistic (LISA) was utilized. LISA is specified as [24]:(2)

Where

*x_i _*and *x_j _*are observations for locations *i *and *j *(with mean *μ*)

*z_i _*= (*x_i _*- *μ*)

*z_j _*= (*x_j _*- *μ*) and

*w_ij _*= spatial weights matrix with values of 0 or 1, based on queen's contiguity.

Rather than using the regular LISA statistic, we implement the LISA with Empirical Bayes (EB) rates. The EB standardization procedure for rates associated with each *i *is detailed more thoroughly in Assuncao and Reis [25] and Bailey and Gatrell [26]. In essence, EB standardization directly standardizes raw rates to obtain a constant variance through a rescaling procedure. Specifically, the original raw rate is replaced with a standardized rate, with mean zero and a standard deviation of one. When this procedure and the resulting rates are combined for use with the LISA statistic, variance instability is reduced - minimizing the chances of spurious inference for the local Moran's *I *test. In this particular application, we substitute roadway miles (instead of population) as the "control" variable for the EB standardization procedure. This is done because areas with commercial establishments - not only the alcohol outlets that we are examining but retail shops, restaurants, shopping centers, etc. - attract substantial non-residential traffic. This can make the local population a less reliable estimate of the population at risk. As a result, the use of roadway miles as the control variable helps account for spatially dense commercial districts throughout Cincinnati.

The resulting statistical output generates a set of categories for significant (*α *= 0.05) blocks in the analysis, representing alcohol outlet agglomerations. In this case, we are particularly interested in the "high-high" category, which represents blocks with high counts of alcohol outlets surrounded by other blocks with similarly high counts of outlets. Due to the use of a first-order queen's contiguity matrix, agglomerations with multiple blocks were contiguous, although there are a few instances of singular blocks that were denoted as high-high due to the use of a spatial lag and the EB standardization procedure.

Fourteen agglomerations of alcohol outlets were identified and denoted as foci for testing the relationship between outlets and simple and aggravated assaults. A representative point is generated for each of the agglomerations to facilitate the assessment of focused clusters. For singular blocks, polygon centroids are generated. For agglomerations with multiple blocks, polygon boundaries are dissolved using a common attribute (e.g., agglomeration ID number) and centroids are generated using newly dissolved polygon boundaries.

To test the potential impact of alcohol outlet agglomerations on violence, a focused test for spatial clustering is used. Substantively different than their more general cluster detection counterparts [27,28], focused tests utilize predefined locales (in our case, the areas of alcohol outlet agglomerations) to explore increased risks for particular outcomes (e.g., disease, violence, etc.), whereas general clustering procedures, such as the LISA, attempt to detect clusters anywhere within the study area. A general null hypothesis for a focused test is simply defined as:

As noted by Waller and Gottway [29], information regarding the magnitude of exposure to the foci may be nonexistent, therefore, increasing distance from the foci is frequently used as a surrogate for decreasing exposure. The focused clustering test used for analysis in this paper is based on Waller et al [30] and is specified as follows:(3)

The score test statistic, *T_sc_*, represents the sum of the difference between the observed (*O_i_*) and expected (*E_i_*) assault counts at each location (*i*,...,*n*), weighted by the exposure to the outlet agglomeration. Inverse distance, 1/*d*_*i*_, is used as the weight for each observation. The null hypothesis for this test is that the observed number of cases in each block is independent, distributed as Poisson random variables with a common assault frequency. The alternative hypothesis is that the observed number of cases in each region is independent, distributed as Poisson random variables where the assault frequency is a proportionally increasing function of exposure. Under the null hypothesis, *T_sc _*should equal 0. Significance is obtained through Monte Carlo simulation (*n *= 999).

## Results

### Cartographic Analysis

**Figure 1:**
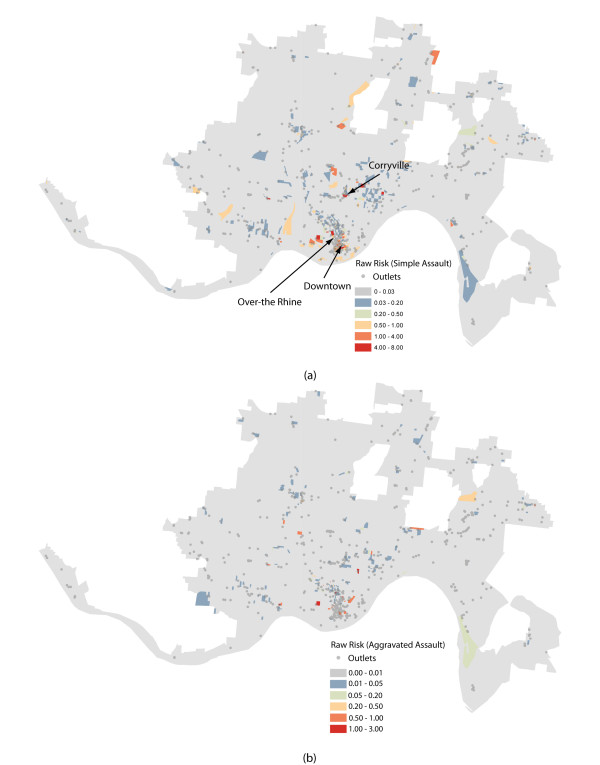
**Raw Risk of Simple and Aggravated Assault by Block: Cincinnati, Ohio (2008)**.

**Figure 2:**
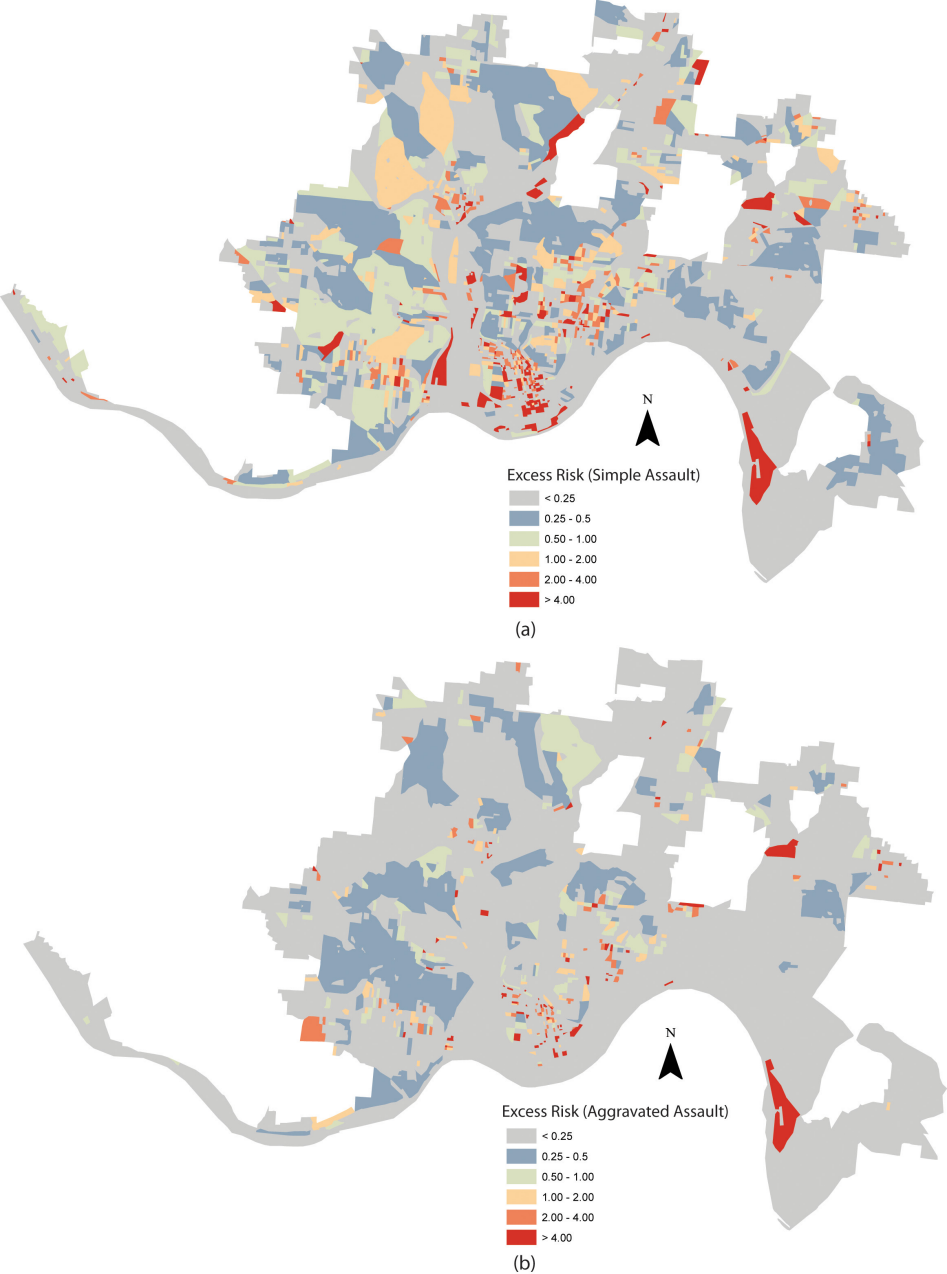
**Excess Risk of Simple and Aggravated Assault by Block: Cincinnati, Ohio (2008)**.

Figure 1a displays all alcohol outlets for the city of Cincinnati, combined with a choropleth map highlighting a population adjusted, raw risk map for simple assault. Figure 1b displays the raw risk map for aggravated assaults, although the outlets are not shown to facilitate a more effective visualization of the assault pattern. Several potentially problematic areas emerge, including downtown and the neighborhoods known as Over-the-Rhine and Corryville. Figure 2 displays the excess risk maps for both assault types in Cincinnati, with patterns largely mimicking Figure 1. In sum, Figures 1 and 2 indicate that several areas within Cincinnati display an elevated level of risk for violence given their local population profile. However, while these maps are informative, they are largely independent statistically from the presence and potential influence of alcohol outlets.

In an effort to better understand the potential role that alcohol outlets play in assaultive violence, a focused clustering test is utilized to explore the difference between observed and expected assault counts, weighted by the degree of exposure to each agglomeration of alcohol outlets.

### Agglomerations of Alcohol Outlets

**Figure 3:**
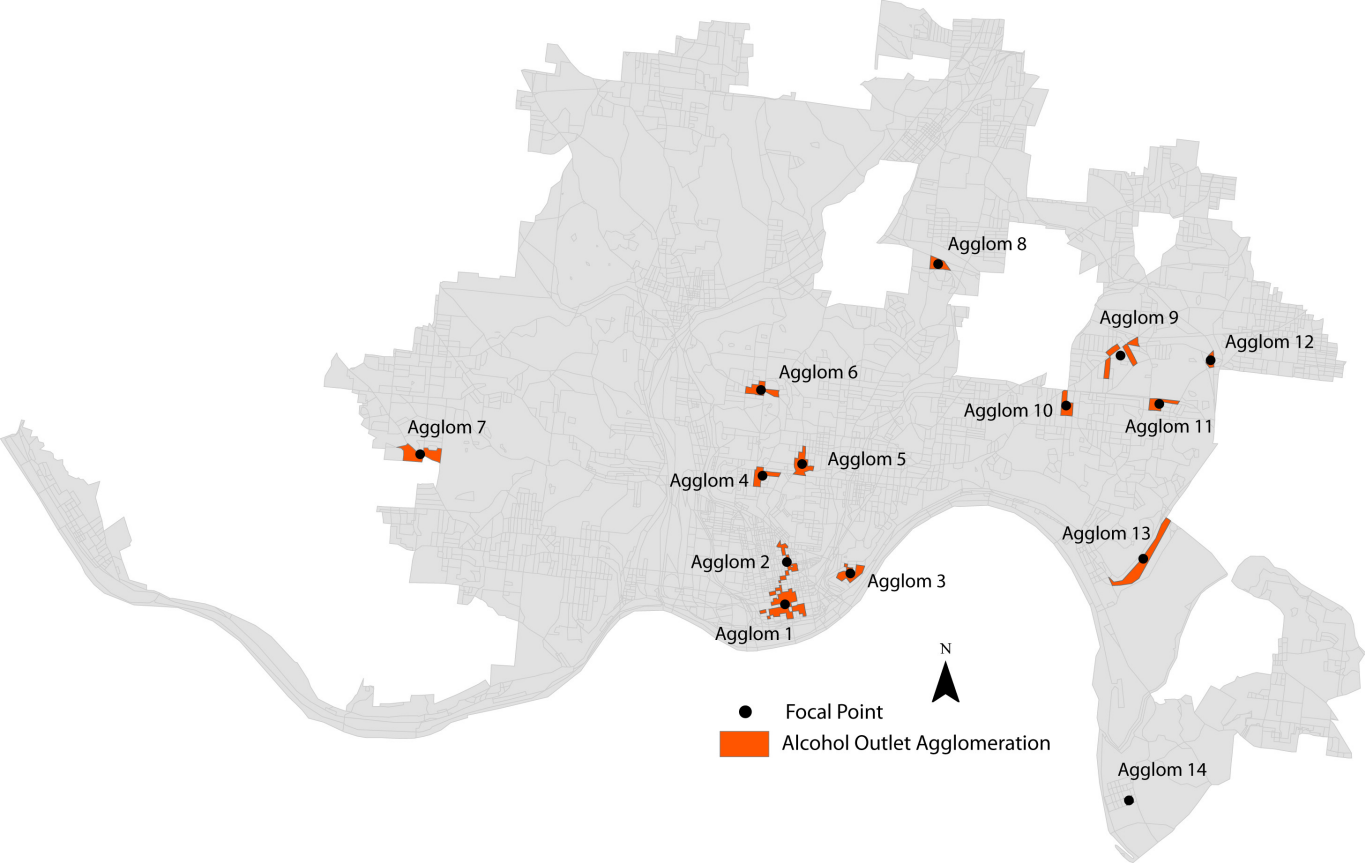
**Alcohol Outlet Agglomerations**.

Outlet agglomerations are identified using the local Moran's *I *test (LISA) for spatial autocorrelation. Figure 3 displays the results, highlighting all blocks in Cincinnati that were assigned to the "high-high" group from the LISA statistic. Again, this represents blocks with high counts of alcohol outlets surrounded by other blocks with similarly high counts of outlets. Because of the use of a spatial lag (queen's contiguity) and the EB standardization process, the actual count of physical outlets does not necessarily represent the lagged count used for the LISA test. Fourteen unique agglomerations were identified and their associated descriptive statistics are displayed in Table 3. It is important to note that all of these agglomerations vary in outlet composition, geographic size, shape, and location. For example, Agglomeration 1, which is located in downtown Cincinnati, contains 58 outlets and is spread across 0.118 square miles of the city. The vast majority of these outlets are restaurants or bars, locales where alcohol is consumed primarily on the premises of the outlets. In contrast, Agglomeration 4, which is located in the central portion of Cincinnati, is geographically small, consisting of a single block with three carry-out shops, four restaurants and four bars.

**Table 3:** Alcohol Outlet Agglomerations

Agglomeration	Total Blocks	Size (square miles)	Total Outlets	Average per Block	Outlet Density*	Type
1	36	0.118	58	1.611	491.525	11 [21] (23) {3}
2	14	0.037	21	1.500	567.568	5 [5] (11) {0}
3	6	0.047	15	2.500	319.149	1 [9] (5) {0}
4	5	0.040	11	2.200	275.000	3 [4] (4) {0}
5	9	0.054	16	1.778	296.296	5 [6] (5) {0}
6	5	0.048	15	3.000	312.500	4 [5] (6) {0}
7	2	0.079	3	1.500	37.975	0 [1] (2) {0}
8	1	0.033	1	1.000	30.303	1 [0] (0) {0}
9	4	0.065	5	1.250	76.923	3 [2] (0) {0}
10	3	0.040	7	2.333	175.000	0 [3] (3) {1}
11	1	0.035	2	2.000	57.143	1 [0] (1) {0}
12	1	0.017	1	1.000	58.824	1 [0] (0) {0}
13	1	0.113	2	2.000	17.699	2 [0] (0) {0}
14	1	0.010	1	1.000	100.000	0 [1] (0) {0}

NOTE: Type count is summarized as follows: off premise [restaurant] (bar) {other)

* Outlet density per square mile

Statistically significant clusters of assaults around the foci

### Alcohol Outlets and Clusters of Violence

**Figure 4:**
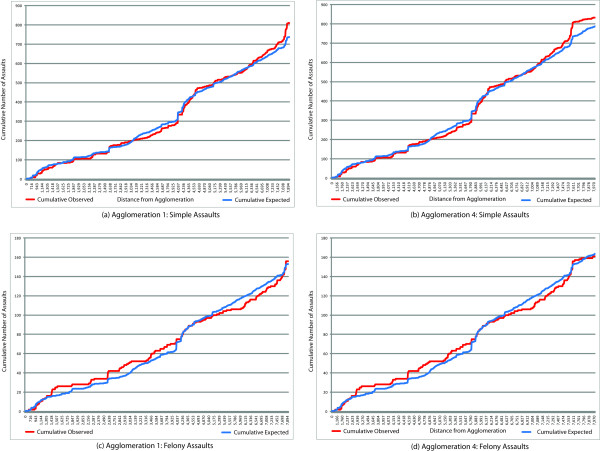
**Observed Versus Expected Assault Counts by Distance from Alcohol Outlet Agglomerations**.

In addition to reporting the test statistic and its associated *p*-value for the focused clustering test, Table 4 also highlights distance thresholds where the observed number of simple assaults exceeds the number of expected assaults with respect to each outlet agglomeration. Three different distance ranges are used, varying with each agglomeration. Distance Range 1 represents the first instance where observed assaults exceed expected assaults for each outlet. For example, consider Agglomeration 1. Not only is it statistically significant, the results suggest that simple assaults begin to cluster at 575 ft. and continue to cluster until 659 ft. While there is no statistically significant clustering between 659 ft. and 1,499 ft. for Agglomeration 1, simple assaults begin to cluster again at a distance of 1,500 ft. and continue until 1,613 ft. (Distance Range 2). Finally, Distance Range 3 indicates that simple assaults cluster between 2,637 ft. and 3,061 ft. for Agglomeration 1. Agglomerations 1-6 are statistically significant for simple assaults, each displaying a different threshold distance where the observed number of assaults exceeds the expected number. Figure 4a provides additional graphical clarification of this process, offering a more global view of the relationship between simple assaults and Agglomeration 1. Red lines represent observed assaults and blue lines represent expected assaults, up to 8,000 ft. When one compares the results between Agglomeration 1 and 4, there are several interesting differences. In addition to the test statistic being much reduced (6.265) for Agglomeration 4, the observed vs. expected cases graph highlights the potential for clustering to occur at different distances in the study area (Figure 4b). For example, Agglomeration 4 does not indicate any clustering until nearly 884 ft., and its third distance range for clusters does not occur until 4,506 ft. Clearly, there is significant variation in the geographic relationship between agglomeration locations and simple assaults in Cincinnati.

**Table 4:** Clustering of Simple Assaults and Associated Distance Ranges

Agglommeration	Test Statistic	p-value	^1^Distance O > E	^2^Distance O > E	^3^Distance O > E
1	38.849	0.001	575 - 659	1500 - 1613	2637 - 3061
2	15.675	0.001	414 - 455	1406 - 1500	2325 - 2785
3	2.716	0.014	696 - 839	3018 - 3232	4528 - 5175
4	6.265	0.001	884 - 948	3180 - 3373	4506 - 4896
5	20.227	0.001	739 - 817	2726 - 3028	4712 - 5289
6	2.242	0.035	943 - 1097	4685 - 4941	6478 - 7381
7	0.651	0.280	-----	-----	-----
8	-0.083	0.506	-----	-----	-----
9	-5.826	1.000	-----	-----	-----
10	-6.572	1.000	-----	-----	-----
11	-1.116	0.851	-----	-----	-----
12	-0.083	0.526	-----	-----	-----
13	0.814	0.205	-----	-----	-----
14	-0.446	0.587	-----	-----	-----

NOTES:

^1 ^First distance range (in feet) where cumulative observed assaults exceeds cumulative expected assaults

^2 ^Second distance range (in feet) where cumulative observed assaults exceeds cumulative expected assaults

^3 ^Third distance range (in feet) where cumulative observed assaults exceeds cumulative expected assaults

The situation is somewhat different for aggravated assaults (Table 5). While Agglomerations 1-5 remain statistically significant, the strength of the test statistic is reduced. For instance, Agglomeration 1 had a *T_sc _*value of 38.84 for simple assaults, but this value falls to 12.30 for aggravated assaults, suggesting fairly moderate reduction in strength. That said, it is interesting to note that the distance thresholds for clustering are nearly the same for aggravated assaults. For example, the distance threshold for observed counts exceeding expected counts for Agglomeration 1 was 575 ft. for simple assaults, extending to 659 ft. However, for aggravated assaults, even though clustering begins at 575 ft., it extends to 751 ft. Clearly, some variation exists between distance thresholds, but the geographic results remains relatively stable. Figures 4c and 4d illustrate these results graphically.

**Table 5:** Clustering of Aggravated Assaults and Associated Distance Ranges

Agglommeration	Test Statistic	p-value	^1^Distance O > E	^2^Distance O > E	^3^Distance O > E
1	12.308	0.001	575 - 751	1197 - 4238	4307 - 4512
2	8.786	0.001	414 - 550	997 - 3856	3906 - 4137
3	1.156	0.014	696 - 1399	2432 - 6050	6077 - 6244
4	2.859	0.007	884 - 1189	2386 - 5920	5957 - 6033
5	6.982	0.001	739 - 1116	1198 - 5243	5299 - 5352
6	-0.501	0.638	-----	-----	-----
7	-1.566	0.897	-----	-----	-----
8	-0.038	0.525	-----	-----	-----
9	-3.319	1.000	-----	-----	-----
10	-3.137	1.000	-----	-----	-----
11	-0.508	0.619	-----	-----	-----
12	-0.038	0.503	-----	-----	-----
13	-0.626	0.667	-----	-----	-----
14	-0.203	0.526	-----	-----	-----

NOTES:

^1 ^First distance range (in feet) where cumulative observed assaults exceeds cumulative expected assaults

^2 ^Second distance range (in feet) where cumulative observed assaults exceeds cumulative expected assaults

^3 ^Third distance range (in feet) where cumulative observed assaults exceeds cumulative expected assaults

## Discussion and Conclusion

Several aspects of this study and its findings warrant consideration in the literature on alcohol outlets and violence. First, the results strongly suggest that assaultive violence frequently clusters near agglomerations of alcohol outlets in Cincinnati. Of the 12 agglomerations tested, both simple and aggravated assaults geographically clustered near five locales. This is an important finding. Not only does it help deepen our understanding of the spatial relationship between alcohol outlets and violence, it provides an additional layer of geographic specificity lacking in previous studies. For instance, while previous work suggests that alcohol outlet density and violence are statistically related [2,14,31], these conclusions are reached at a broader geographic level (e.g., ZIP codes, census tracts and block groups) and tell us little about the actual *spatial relationship *between outlets and violence. We follow up on the careful research carried out by prior scholars that reveals an association between alcohol outlet density and violence by using spatial analytic techniques to identify specific distances where clusters of simple and aggravated assaults begin to form relative to outlet foci, thus adding to the empirical literature on the spatial dynamics of this association [19,20]. Moreover, the use of census blocks reduces the likelihood of pattern obfuscation, a situation that analysts frequently encounter when using more highly aggregated areal units [32].

Second, there is a subtle yet important relationship between the statistically significant agglomerations and their overall geographic composition that supports much of the previous work regarding alcohol outlets and violence. Specifically, areas displaying a higher spatial density of outlets appear to be more prone to clusters of assaultive violence when compared to agglomerations with a lower density of outlets. Reconsider the evidence presented in Table 3. The two most significant clusters of violence are associated with the two most spatially dense agglomerations of outlets. This strongly supports several of the prevalent theories regarding alcohol outlets. For example, several recent studies suggest that the environmental characteristics in and around bars, including staff organization, intoxication of patrons and people remaining around bars after closing can influence levels of violence onsite or nearby [10-12]. Further, the propensity for patrons to hang out around outlets after closing (e.g., nearby parking lots, smoking outside, etc.) may contribute to an increased probability of violent altercations, particularly when social control mechanisms are weakened [33]. Synchronicity of bar closures can also exacerbate these situations [34]. Finally, many off-premise outlets and the areas surrounding them can serve as de-facto taverns in urban areas, where people buy alcohol and congregate for social interaction during consumption [35]. In addition, off-premise outlets often serve neighborhood catchment areas, where people buy alcohol, take it home for consumption and sometimes commit a violent act. As a result, the distance relationship between outlets and violence is multifaceted. In fact, there may be several "critical" distances where the alcohol outlets and violence are related. The empirical evidence presented in this paper certainly suggests that these facets related to distance exist, supporting sound theoretical reasons why violence may cluster around alcohol outlets.

It is important to note that the process used to generate a representative point for outlet agglomerations (i.e., foci) produces a relatively conservative geographic representation of outlet distributions - a single point. In many cases, particularly for agglomerations that consist of multiple blocks, outlets are distributed widely, with many located on or near the periphery of each block. Therefore, because a single point is utilized for generating focused clusters, it is likely that the test statistic is underestimating clustering by inflating the distances between foci and each assault event. This is not always the case, because the distributions of assaults do vary, but it is an important methodological observation to note.

Four important methodological limitations must be acknowledged. As noted previously, there is no way to confirm that the assaults utilized in this analysis were alcohol-related (see [14]). While information regarding the involvement of alcohol in an assault may be recorded as part of the police record of an event, the absence of such information for an event does not mean that alcohol was *not *involved. While this limitation is shared with many previous studies on alcohol outlets and violence, it is not unreasonable to expect that outlet clusters serve to increase non-alcohol-related assaults as well as alcohol-related assaults. A second limitation concerns the statistical power of the focused cluster test and the spatial structure of the data utilized. While census blocks are the most disaggregate spatial units available with basic demographic information, including population, they remain aggregate units. Not surprisingly, population composition and density affect the number of cases expected under the constant risk assumption. As noted by Waller and Gotway [29], areas with more people at risk have higher local sample sizes, which can yield higher power to detect a local increase in relative risk. As a result, there is a propensity for statistical power to exhibit spatial heterogeneities. Put more simply, statistical power and the ability to detect a cluster is contingent on where the cluster occurs.

Third, population was used as the underlying control for measuring risk and calculating expected assaults, yet areas that attract substantial levels of non-residential traffic can exhibit an elevated risk of assault when standardizing by the local population. While this can be problematic, two facets of this study help mitigate these distributional biases. Namely, the use of highly disaggregate units (e.g., census blocks) provides a better snapshot of population distributions. Also, the use of roadway miles instead of population helps to accurately identify clusters of alcohol outlets and better account for commercial districts where outlets may cluster. Finally, social, economic, and demographic factors associated with clusters of assault may also be confounded with outlet agglomerations. For example, alcohol outlets may be clustered in poor and/or disorganized neighborhoods [36]. While our analysis here is focused solely on spatial effects, regardless of such confounders, prior has consistently found an association between the density of alcohol outlets and violence when controlling for a wide range of social, economic, and demographic characteristics.

A final discussion point relates to the policy implications of the results. The spatial distribution of alcohol outlets is not random nor due solely to market forces [36], but is subject to oversight and policy-control mechanisms, both locally and at the state level. For example, the concentration of outlets can be systematically manipulated in multiple ways that other social, economic, and demographic forces cannot. The first and simplest strategy is to limit the number of permits available. This might be accomplished by refusing to issue a new permit when an existing outlet goes out of business, or revoking permits for particularly troublesome outlets. A second, more specific geographic strategy would be to limit the spatial density of permits for an area. Given the results of this study, which suggest that assaultive violence tends to cluster around outlet agglomerations, strategies to reduce the density of alcohol outlets may be a practical option for Cincinnati. Whether this strategy would be a viable one in other communities remains an open research question. Regardless, the existing literature on bars suggests that alcohol-related problems are not distributed evenly. Therefore, as suggested by Madensen and Eck [37], there may be policy mechanisms that can encourage management to make better decisions about controlling on-premise consumption. At the very least this type of analysis can aid law enforcement officials in improving policing efforts by identifying problematic alcohol outlet concentrations, potentially reducing assaultive violence and other alcohol-related problems.

In conclusion, while the results of this paper are compelling, suggesting that assaultive violence clusters around alcohol outlets in Cincinnati, they also serve as a reminder to public health officials, epidemiologists, law enforcement agencies and local governments that we need to develop a better understanding about the specific characteristics of alcohol outlets that may contribute to their association with violence. Thus, while this type of research can answer important theoretical questions, it can also be translational in nature, providing policymakers with potential evidence-based solutions to social problems.

## Competing interests

The authors declare that they have no competing interests.

## Authors' contributions

THG designed the study, conducted the analysis and drafted the manuscript. WAP collaborated in the analysis interpretation and manuscript preparation. All authors read and approved the final manuscript.

## References

[B1] Britt HR Carlin BP Toomey TL Wagenaar AC Neighborhood level spatial analysis of the relationship between alcohol outlet density and criminal violence Environmental and Ecological Statistics 2005 12 411 426 10.1007/s10651-005-1518-3

[B2] Gruenewald PJ Freisthler B Remer L LaScala EA Treno A Ecological models of alcohol outlets and violent assaults: Crime potentials and geospatial analysis Addiction 2006 101 666 677 10.1111/j.1360-0443.2006.01405.x 16669900

[B3] Jones-Webb R McKee P Hannan P Wall M Pham L Erickson D Wagenaar A Alcohol and malt liquor availability and promotion and homicide in inner cities Substance Use & Misuse 2008 43 159 177 10.1080/10826080701690557 18205086

[B4] Scribner RA Mason KE Simonsen NR Theall K Chotalia J Johnson S Schneider SK DeJong W An Ecological Analysis of Alcohol-Outlet Density and Campus-Reported Violence at 32 U.S. Colleges J Stud Alcohol Drugs 2010 71 184 191 20230715 10.15288/jsad.2010.71.184 PMC2841728

[B5] Cunradi CB Mair C Ponicki W Remer L Alcohol outlets, neighborhood characteristics, and intimate partner violence: Ecological analysis of a California city J Urban Health 2011 http://dx.doi.org/10.1007/s11524-011-9549-6 Pre-print (Online First) 10.1007/s11524-011-9549-6 PMC3079039 21347557

[B6] Livingston M A longitudinal analysis of alcohol outlet density and domestic violence Addiction 2011 http://dx.doi.org/10.1111/j.1360-0443.2010.03333.x Pre-print (Early View) 10.1111/j.1360-0443.2010.03333.x 21205052

[B7] Franklin FA LaVeist T Webster DW Pan WK Alcohol Outlets and Violent Crime in Washington D.C West J Emerg Med 2010 11 3 283 290 20882151 PMC2941368

[B8] Lugo W Alcohol and crime: Beyond density Security Journal 2008 21 229 245 10.1057/palgrave.sj.8350056

[B9] Macintyre S Homel R Danger on the dance floor: A study of the interior design, crowding and aggression in nightclubs Crime Prevention Studies 1997 7 91 113

[B10] Graham K Bernards S Osgood DW Wells S Bad nights or bad bars? Multilevel analysis of environmental predictors of aggression in late-night large-capacity bars and clubs Addiction 2006 101 1569 1580 10.1111/j.1360-0443.2006.01608.x 17034436

[B11] Graham K Tremblay PF Wells S Pernanen K Purcell J Jelley J Harm and intent and the nature of aggressive behavior: Measuring naturally-occurring aggression in barroom settings Assessment 2006 13 280 296 10.1177/1073191106288180 16880280

[B12] Graham K Bernards S Osgood DW Homel R Purcell J Guardians and handlers: The role of bar staff in preventing and managing aggression Addiction 2005 100 755 766 10.1111/j.1360-0443.2005.01075.x 15918806

[B13] Peterson RD Krivo LJ Harris MA Disadvantage and neighborhood violent crime: Do local institutions matter? J Res Crime Delinq 2000 37 31 63 10.1177/0022427800037001002

[B14] Livingston M Alcohol outlet density and assault: a spatial analysis Addiction 2008 103 619 628 10.1111/j.1360-0443.2008.02136.x 18339106

[B15] Smith WR Glave S Frazee Davison EL Furthering the integration of routine activity and social disorganization theories: Small units of analysis and the study of street robbery as a diffusion process Criminology 2000 38 489 524 10.1111/j.1745-9125.2000.tb00897.x

[B16] Bromley RDF Nelson AL Alcohol-related crime and disorder across urban space and time: evidence from a British city Geoforum 2002 33 2 239 254 10.1016/S0016-7185(01)00038-0

[B17] Murray RK Roncek DW Measuring diffusion of assaults around bars through radius and adjacency techniques Criminal Justice Review 2008 33 199 220 10.1177/0734016808316777

[B18] Andresen MA Malleson N Testing the stability of crime patterns: Implications for theory and policy J Res Crime Delinq 2011 http://dx.doi.org/10.1177/0022427810384136 Pre-print (Online First)

[B19] Zhu L Gorman DM Horel S Alcohol outlet density and violence: A geospatial analysis Alcohol Alcohol 2004 39 369 375 15208173 10.1093/alcalc/agh062

[B20] Gorman DM Zhu L Horel S Drug 'hot spots", alcohol availability, and violence Drug and Alcohol Review 2005 24 507 513 10.1080/09595230500292946 16361207

[B21] Weisburd D Bernasco W Bruinsma G Putting crime in its place: Units of analysis in geographic criminology 2009 New York: Springer

[B22] Group 1 Software http://www.pbinsight.com

[B23] Ohio Division of Liquor Control http://www.com.ohio.gov/liqr/

[B24] Anselin L Local Indicators of Spatial Association - LISA Geographical Analysis 1995 27 2 93 115

[B25] Assuncao RM Reis EA A new proposal to adjust Moran's I for population density Stat Med 1999 18 2147 2162 10.1002/(SICI)1097-0258(19990830)18:16<2147::AID-SIM179>3.0.CO;2-I 10441770

[B26] Bailey TC Gatrell AC Interactive Spatial Data Analysis 1995 Harlow, Essex: Longman Scientific & Technical

[B27] Aldenderfer MS Blashfield RK Cluster Analysis 1984 Newbury Park: Sage

[B28] Kaufman L Rousseeuw PJ Finding groups in data: An introduction to cluster analysis 1990 New York: Wiley

[B29] Waller LA Gotway CA Applied spatial statistics for public health data 2004 New York: Wiley

[B30] Waller LA Turnbull BW Clark LC Nasca P Chronic disease surveillance and testing of clustering of disease and exposure: Application to leukemia incidence and TCE-contaminated dumpsites in upstate New York Environmetrics 1992 3 281 300 10.1002/env.3170030303

[B31] Gorman DM Speer PW Labouvie EW Subaiya AP Risk of assaultive violence and alcohol availability in New Jersey Am J Public Health 1998 88 97 100 1998 10.2105/AJPH.88.1.97 9584042 PMC1508382

[B32] Grubesic TH Matisziw TC On the use of ZIP codes and ZIP code tabulation areas (ZCTAs) for the spatial analysis of epidemiological data Int J Health Geogr 2006 5 58 10.1186/1476-072X-5-58 17166283 PMC1762013

[B33] Stockwell T Chikritzhs T Do relaxed trading hours for bars and clubs mean more relaxed drinking? A review of international research on the impacts of changes to permitted hours of drinking Crime Prevention and Community Safety 2009 11 153 170 10.1057/cpcs.2009.11

[B34] Humphreys DK Eisner MP Evaluating a natural experiment in alcohol policy: The Licensing Act (2003) and the requirement for attention to implementation Criminol Public Policy 2010 9 41 67 10.1111/j.1745-9133.2010.00609.x

[B35] Block RL Block CR Space, place and crime: Hot spot areas and hot places of liquor-related crime Crime Prevention Studies 1995 4 145 183

[B36] Nielsen AL Hill TD French MT Hernandez MN Racial/ethnic composition, social disorganization, and offsite alcohol availability in San Diego County, California Social Science Research 2010 39 165 175 10.1016/j.ssresearch.2009.04.006 20161391 PMC2782843

[B37] Madensen TD Eck JE Violence in bars: Exploring the impact of place manager decision-making Crime Prevention and Community Control 2008 10 111 125 10.1057/cpcs.2008.2

